# Integrated Mini-Pillar Platform for Wireless Real-Time Cell Monitoring

**DOI:** 10.34133/research.0422

**Published:** 2024-07-24

**Authors:** Yong Luo, Yongchao Song, Jing Wang, Tailin Xu, Xueji Zhang

**Affiliations:** ^1^ Synthetic Biology Research Center, The Institute for Advanced Study (IAS), Shenzhen University, Shenzhen, Guangdong 518060, P. R. China.; ^2^ Beijing Key Laboratory for Bioengineering and Sensing Technology, University of Science and Technology Beijing, Beijing 100083, P. R. China.; ^3^ Research Center for Intelligent and Wearable Technology, College of Textiles and Clothing, State Key Laboratory of Bio-Fibers and Eco-Textiles, Qingdao University, Qingdao 266071, P. R. China.; ^4^ School of Biomedical Engineering, Shenzhen University Health Science Center, Shenzhen University, Shenzhen, Guangdong 518060, P.R. China.

## Abstract

Cell culture as the cornerstone of biotechnology remains a labor-intensive process requiring continuous manual oversight and substantial time investment. In this work, we propose an integrated mini-pillar platform for in situ monitoring of multiple cellular metabolism processes, which achieves media anchoring and cell culture through an arrayed mini-pillar chip. The assembly of polyaniline (PANI)/dendritic gold-modified microelectrode biosensors exhibits high sensitivity (63.55 mV/pH) and excellent interference resistance, enabling real-time acquisition of biosensing signals. We successfully employed such integrated devices to real-time measuring pH variations in multiple cells and real-time monitoring of cell metabolism under drug interventions and to facilitate in situ assisted cultivation of 3-dimensional (3D) cell spheroids. This mini-pillar array-based cell culture platform exhibits excellent biosensing sensitivity and real-time monitoring capability, offering considerable potential for the advancement of biotechnology and medical drug development.

## Introduction

In vitro cell culture aims to simulate in vivo conditions in a controlled artificial environment, meticulously adjusting and monitoring metabolism to ensure cell survival and properties [1–4]. Real-time monitoring of cells is crucial as changes in cellular metabolism effectively reflect the progression of various cellular activities, such as cell growth, differentiation, and drug toxicity [5–7]. Long-term monitoring can effectively assess the evolution of cellular factors over time, reflecting cell vitality and growth dynamics [8–10]. The limited applicability of traditional methods and the demand for precise quantitative monitoring of cellular metabolism have spurred the development of various biosensing techniques [11–13]. For example, integrated optical sensing systems are used for real-time monitoring of inflammatory cytokines in cell culture media [14]. Field’s metal-based electrical impedance spectroscopy is adopted for non-invasive and continuous-flow monitoring of 3-dimensional (3D) cell cultures [15,16]. Nevertheless, the high cost and complexity of operational procedures render these methods unsuitable for routine cell monitoring and hinder their translation into clinical applications [17]. Developing a simple, low-cost, real-time multiplexed monitoring system remains challenging [18,19].

With the advancement of micro-nanofabrication technology, microfluidic chips have been widely developed and constructed, effectively utilized for microdroplet analysis and interface monitoring [20,21]. However, closed micro-chambers and complex structured channels limit their real-time application scenarios [22,23]. As typical open-channel platforms, mini-pillar arrays replace closed microfluidic systems with physical structure, allowing the formation of microdroplets directly with pipettes or droppers for in situ monitoring [24–26]. Furthermore, open-channel platforms can effectively expand to increase throughput and interface with downstream instruments, playing a crucial role in biochemical detection [27,28], material synthesis [29], drug screening [30,31], and cell research [32,33]. In comparison to the high-cost and time-consuming fabrication of microfluidic chips, open-channel platforms emerge as promising platforms with simple structure and high scalability, offering important potential for further development [34–36].

Here, we integrate surface-functionalized microelectrode biosensors to construct an integrated mini-pillar biosensing platform, enabling real-time, in situ monitoring of cell growth processes across various cells. Arrayed mini-pillar chips enable hydrophilic anchoring of culture media and cell array cultivation. Microelectrode biosensors functionalized with polyaniline (PANI) and dendritic gold exhibit excellent linearity (*R*^2^ = 0.999) and high pH sensitivity (63.55 mV/pH) with pH ranging from 6.0 to 8.0. The superior biosensing performance makes it widely applicable, facilitating real-time monitoring of pH changes induced by drug stimuli via the mini-pillar biosensing platform. Long-term in situ monitoring also aids in the auxiliary cultivation of 3D cell spheroids. This straightforward, pH-sensitive, and biocompatible cell culture biosensing platform offers a new approach to monitoring cellular metabolism and holds promise as a powerful tool for in situ cell cultivation.

## Results

**Fig. 1.**
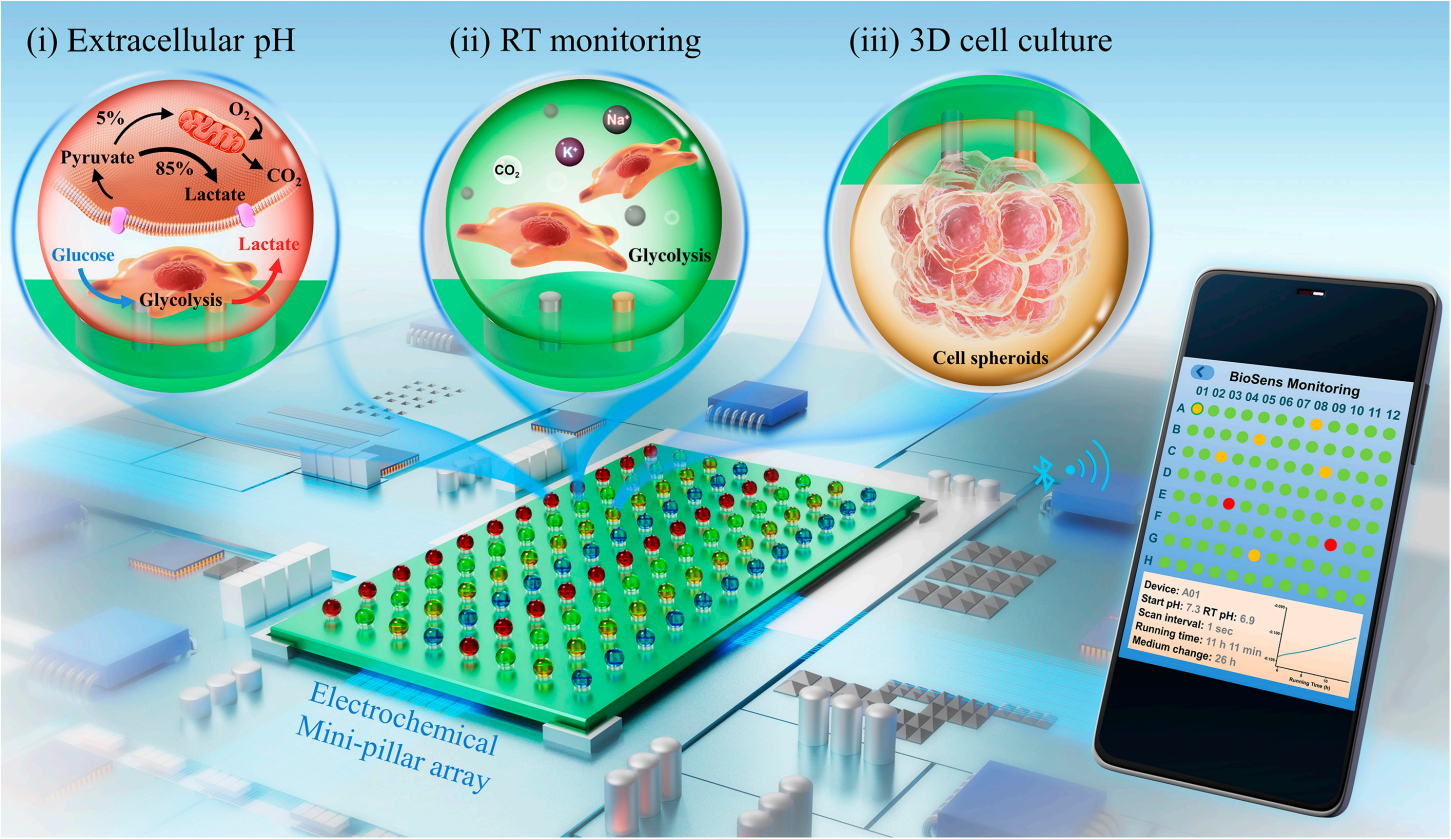
Schematic diagram of an integrated mini-pillar biosensing platform for in situ monitoring of cellular metabolic processes.

Figure 1 is a schematic diagram illustrating the working principle of an integrated mini-pillar platform. The arrayed mini-pillars integrate on-chip microelectrode biosensors to achieve in situ detection of anchored liquid droplets, which can be used for (a) pH measurement of different cells, (b) real-time monitoring of cellular metabolism under drug intervention, and (c) in situ assistance for the cultivation of 3D cell spheroids. The integrated circuit system facilitates signal conversion and output, with an embedded wireless transmission module capable of transmitting biosensor signals to a receiving terminal for remote real-time monitoring. The mini-pillar chip is fabricated using polydimethylsiloxane (PDMS) molding, featuring scalability potential that can be tailored according to application requirements, from single to array configurations.

**Fig. 2.**
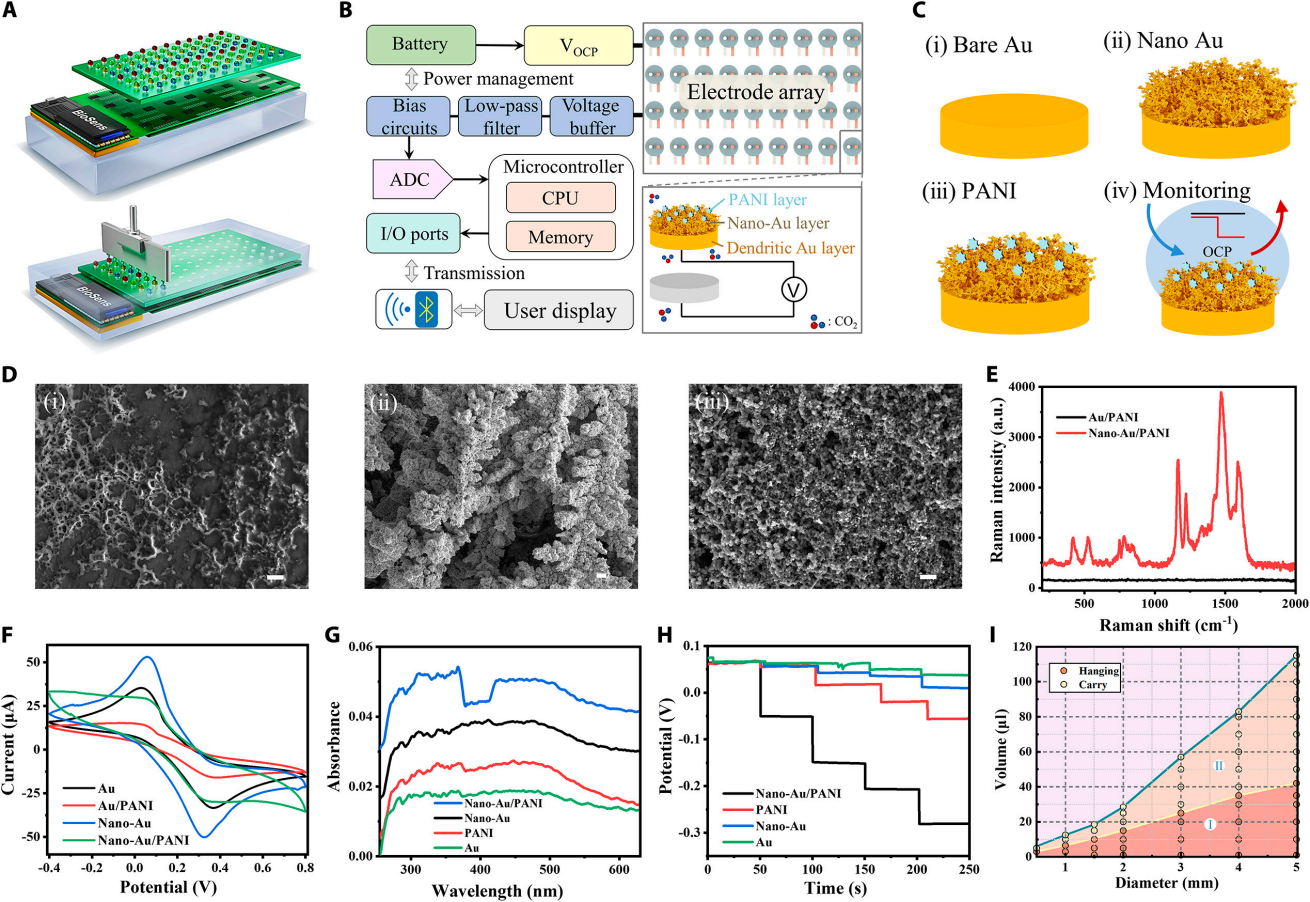
Construction of the integrated biosensing platform and characterization of functional modification. (A) Assembly and working diagram of the integrated mini-pillar biosensing platform. (B) Block diagram for signal acquisition and output in the biosensing platform. (C) Schematic diagram of microelectrode functionalization modification process for signal acquisition. (D) SEM images of functional modification process of microelectrode. Scale bar, 2 μm. (E) Raman spectra characterization of PANI/dendritic gold-modified electrode sensors. (F) CV characterization of electrodes with different modification. (G) UV absorption spectrum characterization of electrodes with different modification. (H) Comparison of biosensing properties of different functionalized microelectrodes. (I) Characterization of the solution carrying capacity of mini-pillar in the integrated biosensing platform.

Considering the limited space and sterility need in the cell culture chamber, an integrated platform was constructed as shown in Fig. 2A [37]. The droplet-carrying mini-pillars adhered to a printed circuit board (PCB), enabling real-time measurement of the potential difference by connecting to the electrochemical biosensing chip. The integration of miniaturized design meets the requirements for conventional cell culture and monitoring biosensing. The boxed diagram in Fig. 2B depicts the driver circuit design of the integrated biosensing chip, which converts and outputs the potential difference measured by the mini-pillars to the receiving terminal. Customized design considerations include amplification and filtering of minute signals, as well as stability requirements for long-term continuous output, ensuring the reliability and accuracy of real-time data. Figure 2C demonstrates the process of modifying high-sensitivity microelectrode sensors: growing dendritic gold structures on the pure gold electrodes, followed by depositing the surface with dopamine thin film to successfully build the working electrode for evaluating pH changes. The scanning electron microscopy (SEM) characterization of the electrode modification process is shown in Fig. 2D, depicting (a) the pure gold substrate, (b) the structure after dendritic gold deposition, and (c) the electrode surface post-modification with polydopamine film. Further confirmation of PANI characteristics was provided through Raman spectroscopy (Fig. 2E), with characteristic Raman peaks located at 1,589, 1,493, 1,360, 1,235, 1,175, 820, 575, and 414 cm^−1^ [38]. Additionally, the significant Raman enhancement peak observed after dendritic gold deposition indicates the presence of more abundant nanogaps, thereby increasing the specific surface area to effectively enhance contact sites and improve the sensitivity of the microelectrode biosensors [39]. Cyclic voltammetry (CV) characterization (Fig. 2F) and ultraviolet–visible (UV–Vis) absorption peak comparison (Fig. 2G) were further utilized to verify the characteristics of PANI. Figure 2H compares the response capabilities of differently modified electrodes to environmental pH changes, demonstrating that our modification can effectively enhance the sensitivity of microelectrode sensors. Additionally, in situ cell culturing requires satisfying loading demands on the culture medium. The mini-pillars we constructed can effectively support microdroplets due to their excellent hydrophobicity and viscoelasticity (Fig. 2I and Fig. S1). Region I indicates that the microcolumns can anchor droplets and rotate 360° (Fig. S2), while region II demonstrates stable load bearing of droplets (Fig. S3). Appropriate sizes according to monitoring needs can be tailored for practical application scenarios.

**Fig. 3.**
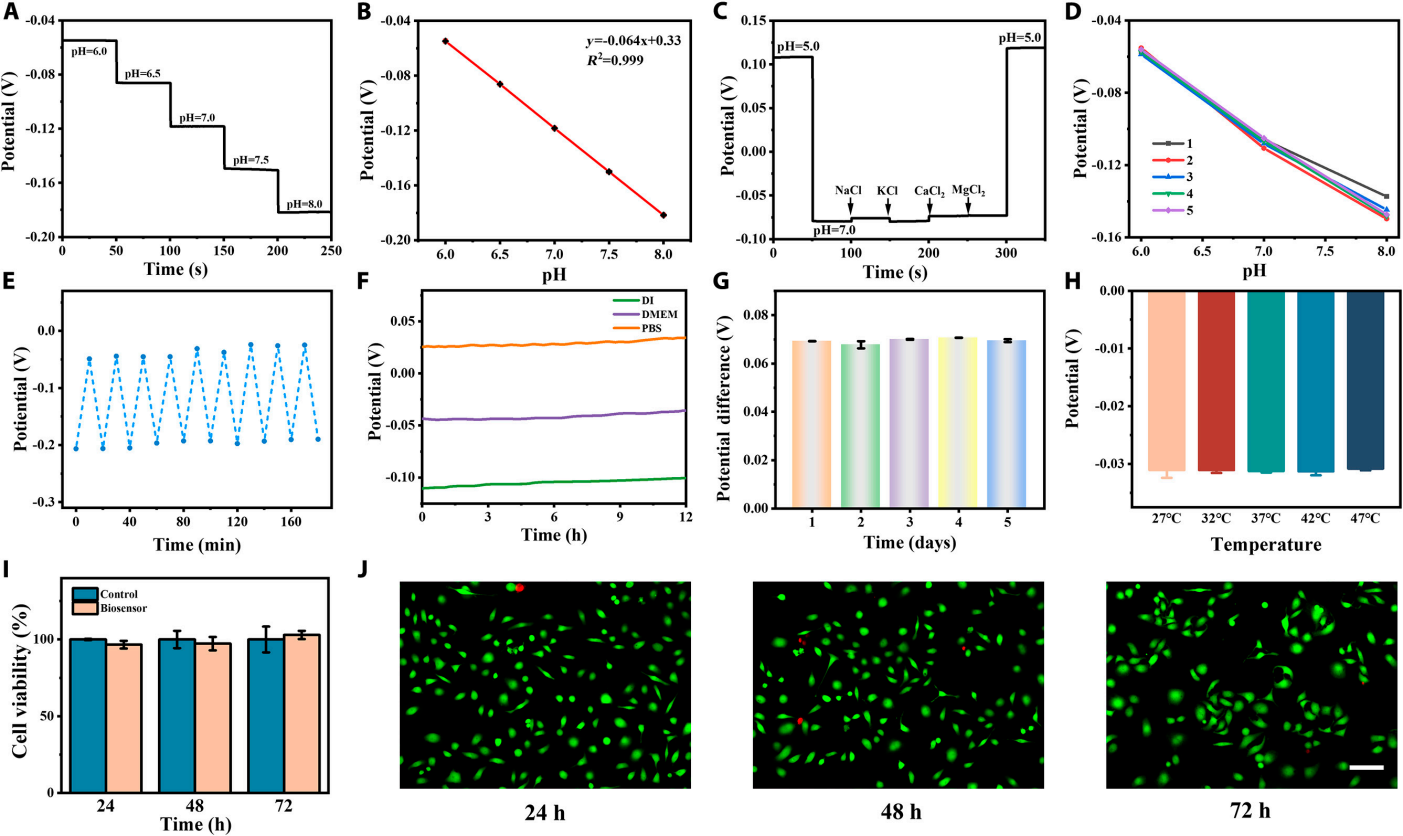
The integrated mini-pillar platform for characterization of pH biosensing performance. (A) Open circuit potential time response of the biosensing platform to different pH solutions. (B) Corresponding linear response curves of open circuit potential of different pH concentrations. (C) Interference tolerance investigation of electrode biosensors toward different ions. (D) Reproducibility verification of different mini-pillar sensor electrodes for pH detection. (E) Verification of long-term reproducibility in different pH solutions. (F) Long-term stability of the mini-pillar biosensing platform at DI water, DMEM, and PBS buffer solution. (G) Comparison of potential difference of microelectrode sensors after different placement durations. (H) Sensitivity comparison of mini-pillar biosensing platform under different temperature conditions. (I) CCK-8 assay of NCM cells for cell viability testing of the biosensors after culturing for 24, 48, and 72 h. (J) Live/dead staining of NCM cells after 24-, 48-, and 72-h incubation with electrode biosensors. Scale bar, 100 μm.

To assess the feasibility of our constructed mini-pillar biosensing platform for real-time cell sensing, we conducted further validation of the sensors’ performance. Based on the possible pH variations of normal cell culture medium (Fig. 3A), we explored the response capability of the sensor within a dynamic range of pH 6 to 8. The corresponding linear relationship between the open-circuit potential difference and pH variation is illustrated in Fig. 3B. The electrodes of the biosensing platform exhibited excellent response sensitivity to pH changes, measuring 63.55 mV/pH, with high linearity (*R*^2^ = 0.999). Selective response to specific analytes in complex environments forms the basis of in situ biosensing [40]. Successive addition of common electrolyte salts and metabolic interferents to the solution did not cause significant interference with real-time potential measurements (Fig. 3C), and the biosensor’s response capability remained consistent. This indicates that the microelectrode biosensor exhibits excellent selectivity and specificity. Reproducibility is another important parameter for the integrated platform in long-term sensing applications [41,42]. Employing scalable manufacturing techniques, such as assembly line operations, can enhance electrode consistency. Figure 3D illustrates the potential changes measured by different electrodes after pH variations, demonstrating the excellent reproducibility of our electrode sensors. Figure 3E presents the relative standard deviation (RSD) of the potential difference calculated as 4.12% from 9 cycles of placing the electrode sensors in 2 pH solutions repeatedly, indicating their robust reproducibility (Fig. S4). Drift is an inevitable issue in potential measurements [43]. Through long-term monitoring of deionized (DI) water, Dulbecco’s modified Eagle’s medium (DMEM), and phosphate-buffered saline (PBS) (Fig. 3F), we observed a drift rate of 5 mV/h for our microelectrode sensors, significantly outperforming current biosensors. Comparative analysis of the response performance of functionalized modified microelectrodes placed for different durations (Fig. 3G) also demonstrates the excellent long-term stability of our sensors. Furthermore, by comparing the sensor response under different temperature conditions, we ruled out the influence of temperature changes on microelectrode performance (Fig. 3H). Finally, we explored the biocompatibility of the mini-pillar platform. Coculturing mini-pillars and electrode materials with human normal colon mucosal cells (NCM-460) cells for 24, 48, or 72 h in CCK-8 experiments revealed no differences in cell viability (Fig. 3I), indicating excellent biocompatibility of our mini-pillar sensors (Fig. 3J). These evaluation results indicate that our mini-pillar platform performs well (Table S1) and has potential for further use in cell pH monitoring.

**Fig. 4.**
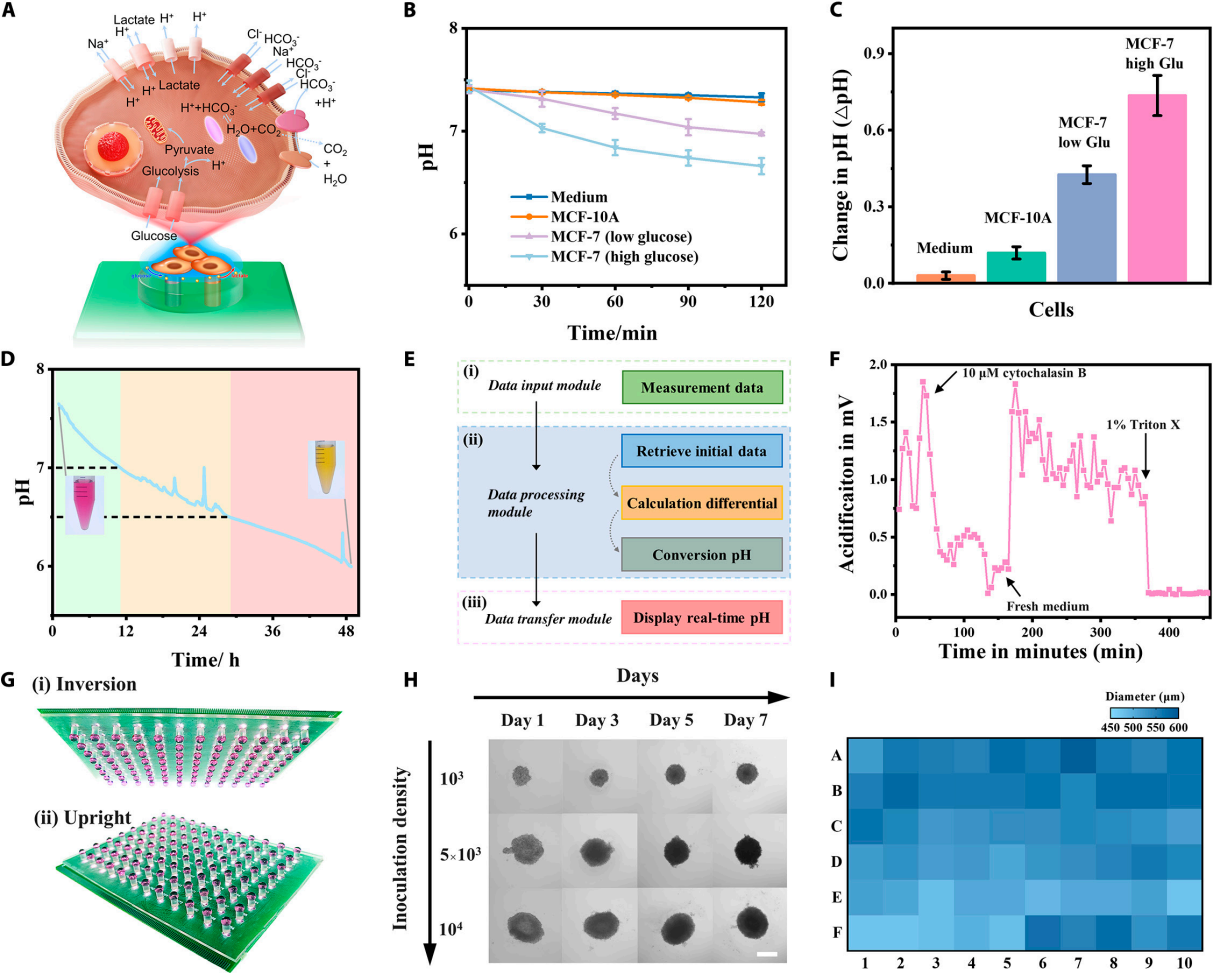
Integrated mini-pillar platform for metabolic monitoring of cells. (A) Mechanisms of protein-mediated cellular metabolic regulation and resulting changes in culture medium pH. (B) Detection of the extracellular acidification (pH change) of normal cells (MCF-10A) and MCF-7 cells at low and high levels of glucose media. (C) pH changes of cancer cells and normal cells in different cell culture media within 120 min. ***P < 0.001 (Student’s *t* test). Error bars represent the SD of the measurement (*n* = 3). (D) Long-term pH monitoring for cell culture. (E) Real-time signal processing mechanism of pH monitoring biosensors. (F) Monitoring the changes of cell metabolism under different drug intervention. (G) 3D cell culture images based on integrated mini-pillar platform. (H) Growth process of 3D cell spheroids with different concentrations over time based on integrated mini-pillar biosensing platform. Scale bar, 500 μm. (I) Heatmap of the diameter distribution of 3D cell spheroids cultivated on integrated mini-pillar platform.

The metabolic mechanisms and homeostasis regulation during cancer cell growth are shown in Fig. 4A. Various membrane proton pumps and transporters expel protons (H^+^) generated by metabolism to the extracellular environment to maintain intracellular pH homeostasis, leading to the gradual acidification of the culture medium [44,45]. Real-time monitoring of pH can thus be used to track the cellular metabolic process. Interstitial space exists between cells and substrates during planar growth, providing conditions for extracellular sensing in our mini-pillar platform [46]. Monitoring results of cell lines cultured in situ on the mini-pillar platform are shown in Fig. 4B. During 120 min of monitoring, the pH change of normal MCF-10A cells was 0.12, while the pH change for the MCF-7 in high-glucose medium was 0.73, indicating a higher pH change caused by cancer cell metabolism compared to normal cells (Fig. 4C). Furthermore, by further reducing the glucose concentration in the cell culture medium, we found that the pH change in cultured MCF-7 cells (low-glucose medium) decreased to 0.42. These results are consistent with previously reported cancer cell metabolism phenomena and further confirm that the abnormal growth and metabolism of cancer cells depend on aerobic glycolysis [47,48]. Our biosensing platform also possesses the capability for long-term monitoring of cell growth. As depicted in Fig. 4D, pH sensing over 48 h effectively covers the normal growth cycle of cells, with the culture medium pH decreasing from 7.65 to 6.03. Real-time analysis of sensing data and output to receiving terminals can assist in prompting actions such as cell medium replacement and passage (Fig. 4E). To validate the monitoring capacity of the platform, we further investigated metabolic rate changes under drug intervention (Fig. 4F). The addition of 10 μM cytochalasin B to cells in a normal growth environment effectively inhibited cellular metabolism. Upon replacement with fresh culture medium, cells resumed normal growth, while the addition of 1% Triton X resulted in the loss of activity in all cells, halting metabolism [49].

Finally, our platform also facilitates the construction of 3D cell spheroids and supports drug toxicity studies. High-throughput culture of homogeneous cell spheroids enables highly reproducible and reliable drug formulation toxicity assessments [50]. Figure 4G depicts physical photographs of 3D cell spheroids in upright and inverted positions. The arrayed mini-pillars can effectively anchor the culture medium and provide nutrients for the spheroids, while the built-in sensing electrodes can monitor metabolic processes and prompt medium replacement in a timely manner. Figure 4H illustrates the changes in cell spheroids over 1 week with different initial concentrations of cell suspensions. Figure 4I provides statistics on the diameter of array-cultivated 3D cell spheroids, with a measured RSD of 5.58%, indicating successful generation of uniformly sized dense cell spheroids on our mini-pillar platform. Besides, microelectrode biosensors enable in situ, label-free metabolic monitoring, offering the potential for time-resolved studies of 3D cell spheroid kinetics and stimulus responses.

## Discussion

In this work, we demonstrate an integrated mini-pillar electrochemical biosensing platform for real-time, in situ, and wireless cell monitoring. Based on template molding and electrochemical deposition, the platform integrates an arrayed droplet anchoring chip, microelectrode biosensors, and signal output system, offering prospects for mass production and practical applications. The constructed microelectrode biosensors exhibit high sensitivity to pH values, along with excellent selectivity, stability, interference resistance, and biocompatibility. The integrated device enables real-time monitoring of extracellular pH fluctuations and explores the regulation of cellular metabolic efficiency by different culture media. Long-term in situ monitoring can provide more effective feedback on cell growth status, such as monitoring changes in cellular metabolism under drug intervention. In general, the integrated mini-pillar platform serves as an auxiliary tool for in situ cell culture, enabling long-term biosensing of cell growth processes and real-time monitoring of various cellular metabolism. Future work will further expand the mini-pillar biosensing platform to simultaneously monitor multiple variables of cell culture characteristics, including pH, dissolved oxygen, lactate, glucose, temperature, and so forth. This will provide an opportunity to enable a more comprehensive assessment of complex cellular physiological changes. Additionally, by combining machine learning techniques, the platform has the potential to analyze and predict metabolic monitoring data. We believe that our mini-pillar platform can serve as an interface for cell culture and real-time monitoring, incorporating with various platforms to unlock more applications in biological and medical research, thereby opening previously unnoticed horizons.

## Materials and Methods

### Construction of integrated high-throughput platform

The integrated mini-pillar biosensing platform comprises a high-throughput mini-pillar array chip, functionalized microelectrodes for real-time sensing signal acquisition, and a sensor control system for signal collection and output. The mini-pillar array platform is constructed using template casting (Fig. S5). A polytetrafluoroethylene (PTFE) template, machined to shape, serves as a mold. PDMS prepolymer and curing agent are mixed at a ratio of 10:1, degassed, poured onto the mold, and cured for 1 h at 40°C. Electrode arrays (Au as working electrodes, Ag as reference/counter electrodes) are embedded into the semi-cured mini-pillars. After curing for 4 h at 60°C, high-throughput mini-pillar arrays, approximately 3 mm in diameter and 1.5 mm in height, are formed. Following detachment, the mini-pillar platform with integrated electrodes is bonded onto a PCB using a 1:1 mixture of prepolymer and cyclohexane adhesive. The corresponding PCB for the mini-pillar array is custom-made by the manufacturer. The sensor control system consists of self-designed PCBs and commercially available electronic components. The open-circuit potential differences measured by the electrode array are converted into pH changes by the sensor control system and transmitted to the signal receiving terminal via a Bluetooth module. A custom 3D-printed plastic cover fits the mini-pillar platform to prevent medium evaporation.

### Construction of pH biosensors

The fingertip electrodes of the integrated circuit board are connected to the electrochemical workstation, where nano-dendritic gold and PANI are sequentially deposited onto the electrodes. Subsequently, 10 μl of specified concentration HAuCl_4_ electrolyte is sequentially added to the mini-pillar array, followed by the initiation of nano-dendritic gold fabrication on the working electrode by setting deposition parameters (the deposition potential is −1 V and the time is 400 s). After 2 depositions, nano-dendritic gold-modified electrochemical sensors are obtained on the mini-pillar sensor. Then, employing a 3-electrode system (Au as the working electrode, Ag as the reference/counter electrode), PANI/dendritic gold-modified electrochemical sensor arrays are electrochemically deposited using 20-CV cycles at a scan rate of 0.1 V/s within a potential range of −0.6 to +1.3 V for further biosensing applications.

### Integrated biosensing platform for measurement

The integrated mini-pillar sensing platform evaluates sensor pH response by recording the potential difference between the PANI/dendritic gold-modified array and Ag/AgCl electrodes. During measurements, the pH of phosphate buffer solutions is adjusted using 0.01 M NaOH and 0.01 M sulfuric acid. The pH of the buffer solutions is quantified using a commercial pH meter (Starter 3100, OHAUS). Ion interference resistance experiments are conducted by titrating chloride solutions containing Na^+^, K^+^, Ca^2+^, and Mg^2+^ into buffer solutions with a pH of 7.0.

## Data Availability

All data needed to evaluate the conclusions in the paper are present in the main text or the Supplementary Materials.

## Supplementary Materials

Supplementary 1 Materials and Methods Figs. S1 to S5 Table S1 References
