# Supplementary material for: Integrated Mini-Pillar Platform for Wireless Real-Time Cell Monitoring
Source: Research (Wash D C). 2024 Jul 24;7:0422. doi: 10.34133/research.0422 (PMC11266812; doi:10.34133/research.0422)
Supplement: Supplementary 1 — Materials and Methods Figs. S1 to S5 Table S1 References [file research.0422.f1.docx]

**Supporting Information**

Integrated Mini-pillar Platform for Wireless Real-time Cell Monitoring

*Yong Luo,^1, 3^Yongchao Song,^4^ Jing Wang,^1^ Tailin Xu,^1, 2,^ * Xueji Zhang^1, 2,^ **

^1^Synthetic Biology Research Center, The Institute for Advanced Study (IAS), Shenzhen University, Shenzhen, Guangdong 518060, P. R. China

^2^School of Biomedical Engineering, Shenzhen University Health Science Center, Shenzhen University, Shenzhen, Guangdong 518060, P.R. China

^3^Beijing Key Laboratory for Bioengineering and Sensing Technology, University of Science and Technology Beijing, Beijing 100083, P. R. China

^4^Research Center for Intelligent and Wearable Technology, College of Textiles and Clothing, State Key Laboratory of Bio-Fibers and Eco-Textiles, Qingdao University, Qingdao 266071, P. R. China

*Corresponding to: xutailin@szu.edu.cn; zhangxueji@szu.edu.cn

1. **Materials and methods**

**1.1 Reagents and materials.**

Chloroauric acid was purchased from Macklin. Potassium chloride, sodium chloride, magnesium chloride and calcium chloride were obtained from Macklin. Sodium hydroxide was purchased from Shanghai Yien Chemical Technology Co., Ltd. Sulfuric acid was purchased from Xilong Scientific Co., Ltd. The Polydimethylsiloxane (PDMS) was purchased from Dow Chemical (Malaysia) Sdn. Bhd. Phosphate buffer saline (PBS) was purchased from Sigma. Fetal bovine serum (FBS), and Trypsin were purchased from GIBCO. Penicillin-Streptomycin was purchased from GIBCO. Dulbecco’s modified Eagle’s medium (DMEM) (high glucose, low glucose) were purchased from GIBCO. Calcein-AM/PI Double Stain Kit (Cat No. 40747ES) was purchased from Yeasen Biotechnology (Shanghai) Co., Ltd. Cell Counting Kit-8 (Cat No. 40203ES) was purchased from Yeasen Biotechnology (Shanghai) Co., Ltd. Triton X-100, Molecular Biology Grade (Cat No. 20107ES20) was purchased from Yeasen Biotechnology (Shanghai) Co., Ltd. Cytochalasin B was purchased from Aladdin. Methyl cellulose was purchased from Macklin. The polytetrafluoroethylene (PTFE) template with array was custom made from Beijing Zhongjingkeyi Technology Co., Ltd, China. All chemicals were used without any further purification and prepared by dilution using ultrapure water (Milli-Q, 18.2 MΩ•cm) otherwise specified. All experiments were carried out at room temperature (25 °C).

**1.2 Experimental setup.**

The modified electrodes were characterized for morphology using High Resolution Scanning Electron Microscope (APREO S, Thermo Scientific™). The laser confocal Raman Spectrometer (InVia, Renishaw, England) connected to a telephoto objective (50×, Leica) was used for Surface Enhanced Raman Scattering sensing (532 nm laser excitation). The Nikon Eclipse Ni microscope, coupled with a 4X objective and a Nikon DS-Ri2 microscope camera were used for 3D spheres real-time observation. UV-visible spectroscopy was conducted using a UV-2600i spectrophotometer (Shimadzu) to record. Cell fluorescence imaging was achieved by Uitra-high resolution confocal microscopy (LSM880, ZEISS). Electrochemical deposition was carried out using an electrochemical workstation (CHI 660, Shanghai Chenhua Instrument). Cell viability was assessed using an enzyme labeling reader. The average length of 3D spheres was counted using ImageJ software.

Thawed liver cancer MCF-7 cells were dispersed in complete medium containing 10% fetal bovine serum (FBS) and Dulbecco's Modified Eagle's Medium (DMEM, Gibco), then cultured in a CO2 incubator at 37°C. Passaging was conducted following the experimental protocol provided by American Type Culture Collection (ATCC). Adherent cells were detached using trypsin digestion (1000 rpm, 3 min), diluted to a concentration of 1.0 × 10^5^ cells/mL, and seeded onto the mini-pillar array with 20 μL. Cell growth was monitored in real-time by measuring the potential changes of the electrode under optimal cell culture conditions.

**1.3 Cell viability**

The cell suspension was distributed into a 96-well plate at a concentration of 10^4^ cells/mL, with experimental and control groups designated. The experimental group was supplemented with electrode materials and co-cultured for 24 hours, 48 hours and 72 hours. Subsequently, the cell viability was further measured using a CCK-8 assay kit. The CCK-8 assay was performed following the manufacture’s recommendation and the data were presented as percentage in respect to the cells. Absorbance of each well was measured using enzyme labeling reader, with the wavelength set to 450 nm.

**1.4 3D cell spheres culture**

Prepare cell suspension from adherent cells using the same method as 1.3 section. Different cell densities can yield 3D cell spheres of various sizes; therefore, cell concentration was measured using a cell counter and diluted with fresh complete medium to the desired concentration. Inoculate 20 µL of cell suspension onto each mini-pillar of the integrated biosensing platform, followed by incubation at 37°C and 5% CO_2_. The open circuit potential signals monitored pH changes through the biosensing platform, while microscopy was employed to observe cell shape changes. During media replacement, 50% of the spent medium was replaced with 50% fresh complete medium to facilitate further cultivation.

**1.5 pH calculation**

The real-time open-circuit potential (OCP) measurements were converted to pH values by Equation (1) as follows:

  $\text{pH}=\frac{E_{0}-E}{0.064}+p_{0}$ (1)

Where *E* is the real-time open-circuit potential measured by the mini-pillar sensing platform, *E_0_* is the potential value measured during initial calibration, and *p_0_* is the initial pH corresponding to *E_0_* (averaged from three repeated measurements).

1. **Supporting Figures**

**
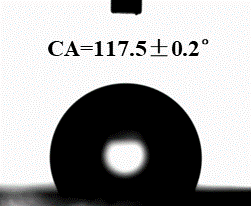
**

**Fig. S1** Contact Angle measurement of mini-pillar platform.

**
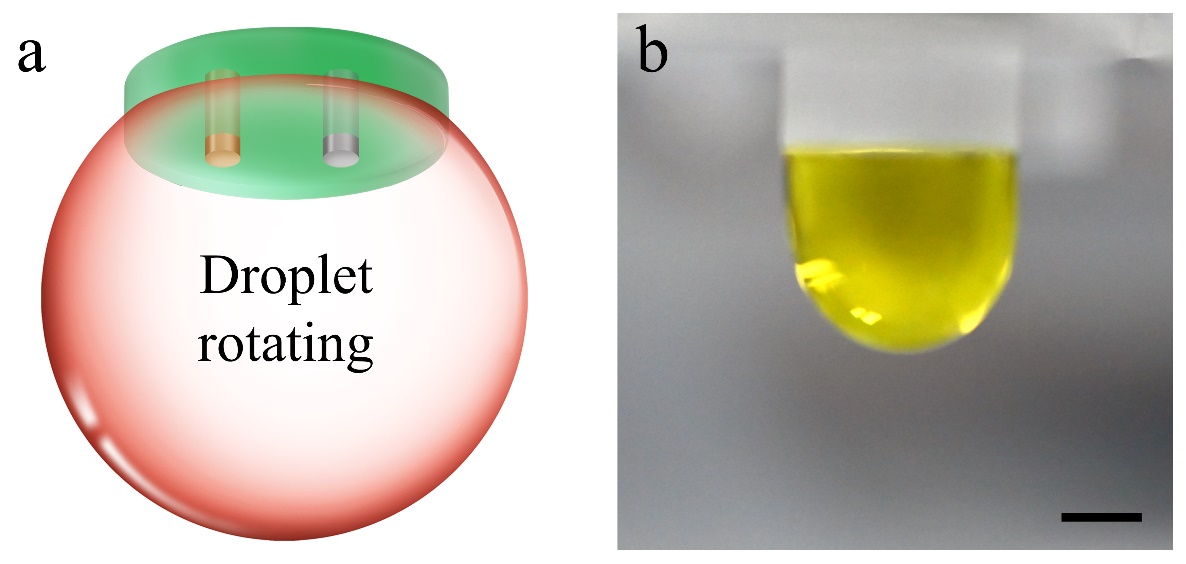
**

**Fig. S2** Schematic diagram (a) and actual photograph (b) of the mini-pillar anchoring the microdroplet and rotating 180 degrees. Scale bar: 1cm.


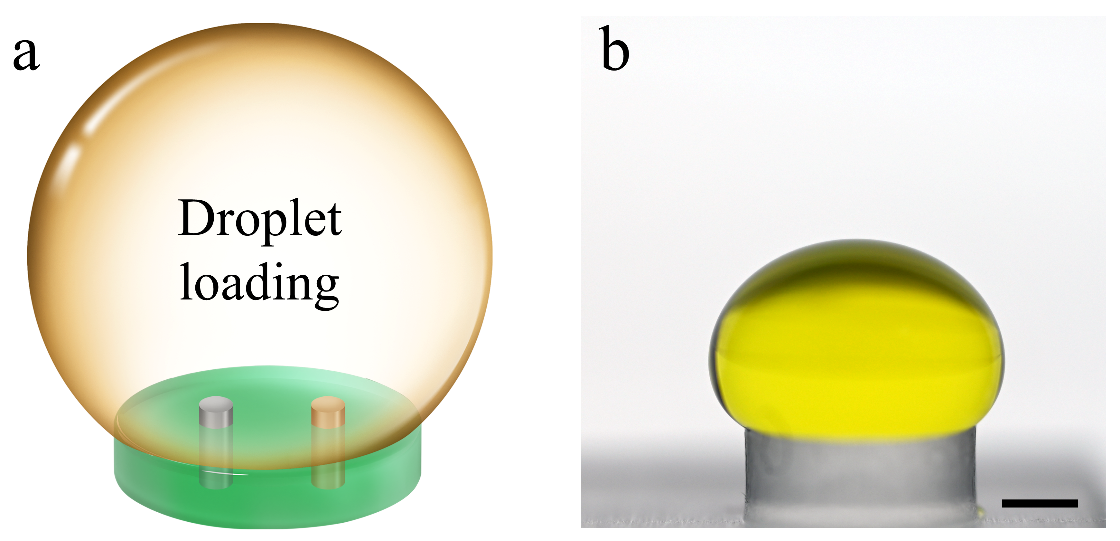


**Fig. S3** Schematic diagram (a) and an actual photograph (b) of the mini-pillar stabilizing the microdroplet. Scale bar: 1cm.

**
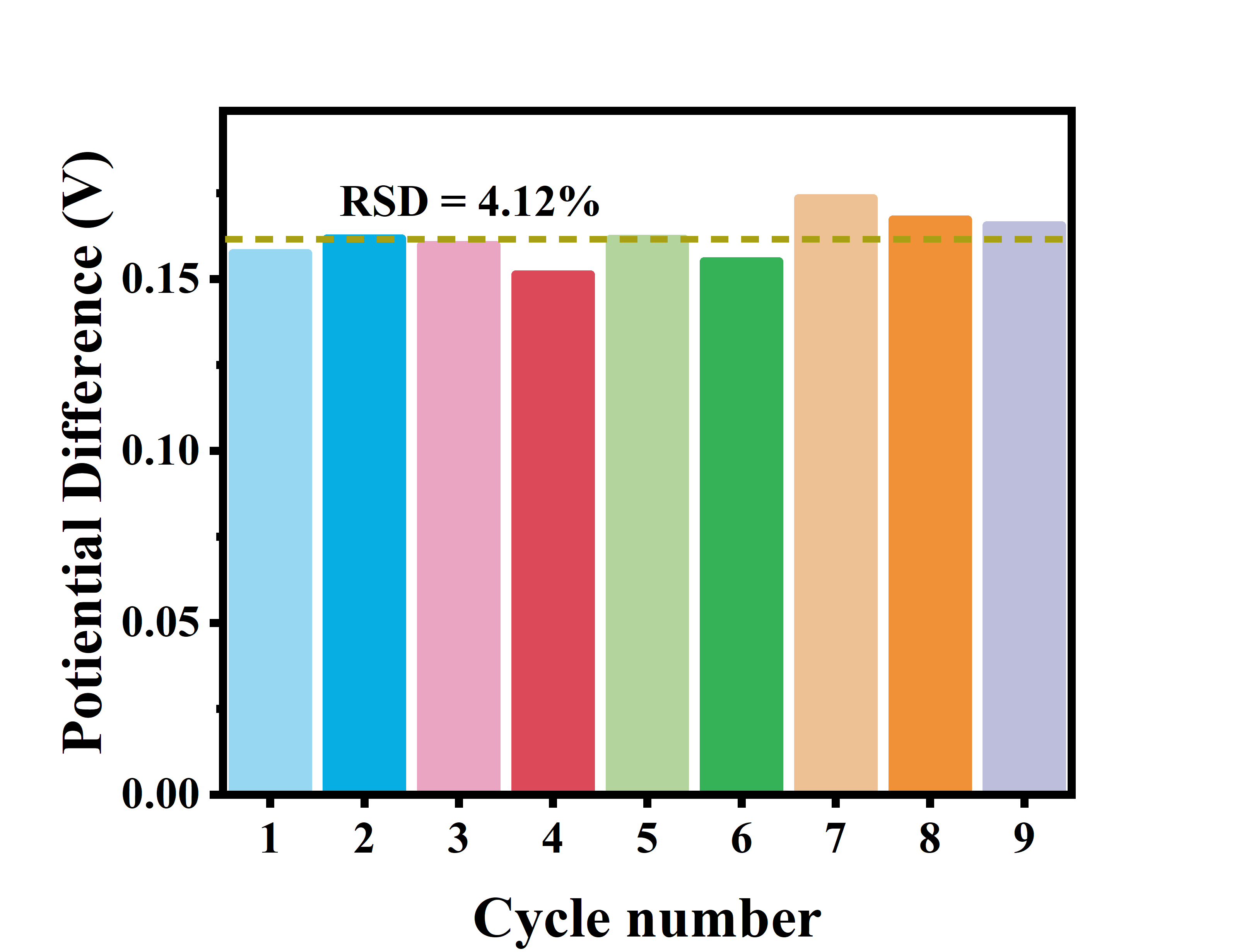
**

**Fig. S4** The reproducibility and robustness assessment of the potential difference for the electrode biosensors.

**
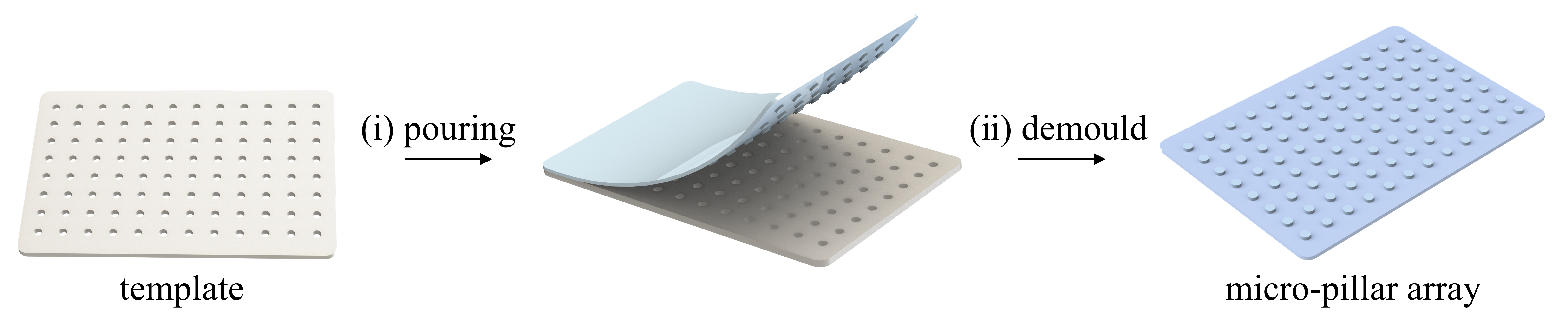
**

**Fig. S5** Schematic of the fabrication steps for constructing the mini-pillar array using the template casting.

1. **Supporting Table**

**Table S1. Comparison of the biosensing platform with other biosensors.**

| **Materials** | **Method** | **Detection range (pH)** | | **Integrated monitoring** | **Detection sensitivity** | **Reference** | |
| --- | --- | --- | --- | --- | --- | --- | --- |
| Graphene oxide/syringaldazine | CV | 5.65-7.49 | No | | 60 mV/pH | | [1] |
| Iridium Oxide | CV | 1-13 | No | | 60.23 mV/pH | | [2] |
| Ubiquinone/ carbon nanotube | CV | 6-9 | No | | 0.10 /pH | | [3] |
| SiNPs | Colorimetric | 5-10 | No | | 0.5 /pH | | [4] |
| IrOx/screen-printed | CV | 1-9 | Yes | | 74.3 mV/pH | | [5] |
| Iridium oxide | OCP | 3.5-11 | No | | 55.9 mV/pH | | [6] |
| IrO_2_/gold | OCP | 4-10 | Yes | | 69.9 mV/pH | | [7] |
| Tungsten | PHAIR | 4-10 | Yes | | 42.7 mV/pH | | [8] |
| Polyaniline/ dendritic gold | OCP | 6-8 | Yes | | 63.55 mV/pH | | Our work |

**References**

1. Munteanu, R. E.; Stanica, L.; Gheorghiu, M.; Gaspar, S., Measurement of the Extracellular pH of Adherently Growing Mammalian Cells with High Spatial Resolution Using a Voltammetric pH Microsensor. *Anal. Chem.* **2018,** *90*, 6899-6905.

2. Xi, Y.; Guo, Z.; Wang, L.; Xu, Q.; Ruan, T.; Liu, J., Fabrication and Characterization of Iridium Oxide pH Microelectrodes Based on Sputter Deposition Method. *Sensors* **2021,** *21*, 4996.

3. McBeth, C.; Dughaishi, R. A.; Paterson, A.; Sharp, D., Ubiquinone modified printed carbon electrodes for cell culture pH monitoring. *Biosens. Bioelectron.* **2018,** *113*, 46-51.

4. Na, M.; Han, Y.; Chen, Y.; Ma, S.; Liu, J.; Chen, X., Synthesis of Silicon Nanoparticles Emitting Yellow-Green Fluorescence for Visualization of pH Change and Determination of Intracellular pH of Living Cells. *Anal. Chem.* **2021,** *93*, 5185-5193.

5. Cheng, C.; Wu, Y.; Li, X.; An, Z.; Lu, Y.; Zhang, F.; Su, B.; Liu, Q., A wireless, ingestible pH sensing capsule system based on iridium oxide for monitoring gastrointestinal health. *Sensor. Actuat. B-Chem.* **2021,** *349*, 130781.

6. Xiao, W.; Dong, Q., Iridium oxide and cobalt hydroxide microfluidic-based potentiometric pH sensor. *Mikrochim Acta* **2023,** *190*, 457.

7. Chung, H.-J.; Sulkin, M. S.; Kim, J.-S.; Goudeseune, C.; Chao, H.-Y.; Song, J. W.; Yang, S. Y.; Hsu, Y.-Y.; Ghaffari, R.; Efimov, I. R.; Rogers, J. A., Stretchable, Multiplexed pH Sensors With Demonstrations on Rabbit and Human Hearts Undergoing Ischemia. *Adv. Healthc. Mater.* **2014,** *3*, 59-68.

8. Dabaghi, M.; Saraei, N.; Xu, G.; Chandiramohan, A.; Yeung, J.; Nguyen, J. P.; Vukmirovic, M.; Selvaganapathy, P. R.; Hirota, J. A., PHAIR: a biosensor for pH measurement in air-liquid interface cell culture. *Sci. Rep.* **2021,** *11*, 3477.
